# Suppressor of cytokine signaling 2 is associated with growth impairment in pediatric chronic kidney disease

**DOI:** 10.1007/s00467-025-07029-0

**Published:** 2025-11-28

**Authors:** Anna Deja, Beata Leszczyńska, Nikola Materny, Maja Wujec, Anna Stelmaszczyk-Emmel, Małgorzata Pańczyk-Tomaszewska

**Affiliations:** 1 Department of Pediatrics and Nephrology, Medical University of Warsaw, Warsaw, Poland; 2 Doctoral School, Medical University of Warsaw, Warsaw, Poland; 3 Student Scientific Group, Department of Pediatrics and Nephrology, Medical University of Warsaw, Warsaw, Poland; 4 Department of Laboratory Diagnostics and Clinical Immunology Developmental Age, Medical University of Warsaw, Warsaw, Poland

**Keywords:** Growth impairment, Chronic kidney disease, SOCS2, Children, Subclinical inflammation

## Abstract

**Background:**

Growth failure is a common and serious complication of chronic kidney disease (CKD) in children, resulting from numerous factors, including growth hormone (GH) resistance. Suppressor of cytokine signaling 2 (SOCS2), a negative regulator of GH signaling, has been implicated in growth regulation but has not been previously studied in pediatric CKD.

**Methods:**

In this cross-sectional study, we assessed serum SOCS2 concentrations in 55 children with CKD (stages 2–5) and 27 age- and sex-matched healthy controls using a high-sensitivity ELISA. We evaluated anthropometric parameters, kidney function, and biochemical markers. Associations between SOCS2 levels and growth (height standard deviation scores, HtSDS) were analyzed using correlation and multivariate regression models.

**Results:**

SOCS2 levels were significantly higher in children with CKD compared to controls (median 1526.5 vs. 1294.6 pg/ml, *p* < 0.001). In patients with CKD, SOCS2 negatively correlated with HtSDS (*r* =  −0.30, *p* = 0.029). In multivariate analysis, SOCS2 was an independent predictor of lower HtSDS alongside eGFR. SOCS2 concentrations were higher in short-statured children, and ROC analysis showed acceptable diagnostic performance for predicting short stature (AUC 0.78, 95% CI 0.64–0.91; 79.5% sensitivity, 68.8% specificity).

**Conclusions:**

Circulating SOCS2 levels are elevated in pediatric CKD and independently associated with growth impairment, suggesting its potential link to GH resistance, which is typical of CKD. SOCS2 might be a potential new marker of growth retardation. Further longitudinal studies are needed to explore its predictive value for longitudinal growth and GH treatment response.

**Graphical abstract:**

**Figure:**
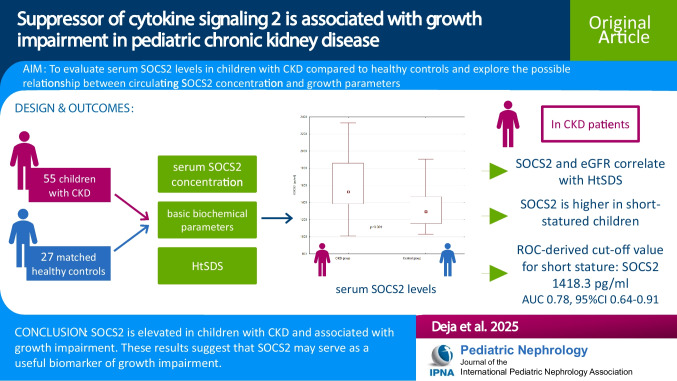
A higher resolution version of the Graphical abstract is available as Supplementary information

**Supplementary Information:**

The online version contains supplementary material available at 10.1007/s00467-025-07029-0.

## Introduction

Chronic kidney disease (CKD) in children is a progressive condition associated with a number of systemic complications, among which growth failure is one of the most prevalent [1, 2]. Despite advances in nutrition, hormonal, and kidney replacement therapies, many children with CKD fail to reach their genetic growth potential [3], which negatively affects their quality of life and long-term health outcomes [4]. The underlying mechanisms of CKD-related growth impairment are multifactorial, involving nutritional deficits, metabolic acidosis, inflammation, and resistance to growth hormone (GH) signaling [5].

The suppressor of cytokine signaling 2 (SOCS2) protein plays an important role in modulating GH and cytokine signaling through the JAK-STAT pathway, acting as a negative regulator of GH receptor signaling [6, 7] (Supplementary Fig. 1). Previous studies on murine models have shown that overexpression of SOCS2 leads to growth suppression, while its deficiency results in increased longitudinal growth [8]. Although SOCS2 is an intracellular protein and has primarily been studied as such, recent advances in assay sensitivity have allowed for its detection in human serum using high-sensitivity ELISA kits [9, 10], opening the possibility of assessing systemic SOCS2 expression as a biomarker. To the best of our knowledge, SOCS2 serum levels have not yet been studied in the context of pediatric CKD and growth retardation.

The aim of this study was to evaluate serum SOCS2 levels in children with CKD compared to healthy controls, and to assess the possible relationship between circulating SOCS2 concentration and growth parameters. We investigated whether elevated serum SOCS2 could be associated with growth retardation in pediatric CKD.

## Materials and methods

### Study population

This was a cross-sectional undertaking. Patients hospitalized in a tertiary referral pediatric nephrology center were evaluated and recruited for the study. The inclusion criteria were (1) CKD stages II–V, diagnosed in accordance with the KDIGO definition [11], and (2) metrical age between 2 and 18 years. The exclusion criteria were (1) history of kidney or other organ transplantation, (2) glucocorticoid therapy during the preceding 3 months (to minimize the chances of its influence on cytokines and SOCS2), (3) lack of consent, (4) active infection at recruitment time, and (5) confirmed short stature of different origin than CKD (including Turner, Prader-Willi, and Down syndromes, skeletal dysplasias, somatotropic or combined pituitary deficiency, children diagnosed with intrauterine growth restriction or small for gestational age, and other confirmed active or unstable conditions that could influence growth). In all, 55 patients with CKD were recruited for the study. The control group consisted of 27 healthy volunteers who were age- and sex-matched to the study group. Because to date there are no data on serum SOCS2 levels in children, we based the sample size approximation on the literature concerning proinflammatory cytokines in children with CKD and taking into account our preliminary study (unpublished data, see Supplementary Fig. 2), with statistical power 0.80, *p* < 0.05, the sample size for the CKD group was assessed to be 50.

### Ethical considerations

An approval from the Local Bioethics Committee of the Medical University of Warsaw was obtained (approval no. KB/151/2020). All procedures involving human participants were run in accordance with the ethical standards of the institutional research committee and were performed in accordance with the declaration of Helsinki and its later amendments. All patients’ legal representatives and patients aged 16 years or older signed informed consent to participate in the study.

### Clinical and anthropometric parameters

For each patient, the examinations were performed on the same day as blood sampling. Among clinical parameters, we assessed age, sex, CKD stage, and primary kidney disease. The basic anthropometric parameters included height and body mass index (BMI). As standard clinical practice, we used WHO Child Growth Standards for assessing anthropometric features in children younger than 3 years of age [12], and the Polish OLA-OLAF growth references for those aged 3 years and above [13, 14]. As this study involved children aged 2 and above, the SDS values are calculated based on the WHO Child Growth Standards to ensure consistency of this continuous parameter. Due to discrepancies between the growth charts [15], we applied the standard practice strategy for assessing short stature, defining it as HtSDS below  − 2.0 based on the appropriate growth chart as mentioned above (OLA-OLAF or WHO depending on age).

### SOCS2 concentration

In all study participants, blood was collected in the morning. The samples were allowed to clot, then promptly centrifuged under controlled conditions. Serum was separated and frozen at  −80 °C for further testing. The samples were thawed immediately before testing. The concentration of SOCS2 [pg/ml] was determined using the ELISA (enzyme-linked immunosorbent assay) method, with the AssayGenie Human SOCS2 kit (catalogue number HUFI01287), in accordance with the manufacturer’s protocol.

### Laboratory testing

The remaining blood samples were immediately subjected to laboratory testing, using a uniform, standardized method. We assessed the following: complete blood count (CBC) (hemoglobin [g/dl] and inflammation parameters: neutrophil, lymphocyte, and platelet counts, mean platelet volume [fL]); standard CKD-MBD markers: total calcium [mg/dl], phosphate [mg/dl], alkaline phosphatase (ALP) [IU/l], parathyroid hormone (PTH) [pg/ml]; serum total protein [g/dl] and albumin [g/dl] levels; blood gases analysis (base excess [mmol/l] and bicarbonate [mmol/l]); kidney function markers: urea [mg/dl] and creatinine [mg/dl]; insulin-like growth factor 1 (IGF1) [ng/ml].

eGFR was calculated using the bedside Schwartz formula based on serum creatinine [16]. We also calculated the CBC-derived inflammatory parameters: neutrophil-to-lymphocyte ratio (NLR) and platelet-to-lymphocyte ratio (PLR).

### Statistical analysis

Statistical analysis was performed using Dell Statistica 13.3 PL software. The normality of data was checked using the Shapiro–Wilk test. The results are expressed as median (IQR). The following statistical tests were used: Mann–Whitney *U* test, Spearman’s rank correlation, and *χ*^2^ test. We applied receiver operating characteristics (ROC) analysis to calculate the area under the curve (AUC), and the cut-off value was identified with Youden’s index. We performed multivariate analysis using the general step-wise regression models. The variables correlating with each other with *r* > 0.60 were excluded to avoid collinearity. Variables were included in the final model with *p* < 0.050. The results of multivariate analyses were expressed as *R*^2^, *β*, confidence interval (CI), and *p*-value. All results were considered statistically significant, with *p*-values < 0.050. Descriptive statistics were performed for the entire population as well as separately for the CKD and control groups. Comparative statistics assessed differences between CKD and control groups. Multivariate analyses concerning HtSDS and SOCS2 levels, as well as the ROC analysis for predicting short stature were performed for the CKD group.

## Results

### Characteristics of the study group

Clinical and anthropometric characteristics of the examined group are shown in Table 1. Patients with CKD did not differ from the control group regarding age or sex distribution. Around half of the patients with CKD (*n* = 28, 50.9%) had mild-to-moderate CKD (stages 2 and 3), and the remaining patients (*n* = 27, 49.1%) had severe kidney function reduction (stages 4 and 5). Among children with CKD stage 5, seven required chronic dialysis (three hemodialysis, four peritoneal dialysis). CAKUT was the leading cause of CKD in our population (*n* = 28, 50.9%). Fifteen children in the CKD group (27.3%) and none in the control group were born prematurely. Three children in the CKD group had a history of past high-dose glucocorticoid use (defined as a dose equivalent to either ≥ 2 mg/kg or ≥ 20 mg/day of prednisone or equivalent for persons who weigh > 10 kg, when administered for ≥ 14 consecutive days [17]).

**Table 1:** Clinical and anthropometric characteristics of the examined group

Parameter	CKD patients (*n* = 55)	Control group (*n* = 27)	*P*
Sex (*n*, female/male)	14/41	8/19	0.689
Age (years)	7.4 (3.9–12.3)	7 (5.0–9.3)	0.875
Height SDS	− 1.7 (− 2.4 to − 1.1)	0.7 (0.1–1.6)	< 0.001
BMI SDS	− 0.31 (− 1.22–0.64)	0.20 (− 0.41–0.78)	0.174
CKD stage [*n* (%)]			
2	5 (9.1%)	-	-
3	23 (41.8%)		
4	16 (29.1%)		
5 [5D]	11 (20.0%) [7, (12.7%)]		
Primary kidney disease [*n* (%)]			
CAKUT	28 (50.9%)	-	-
Cystic kidney disease	7 (12.7%)		
Hereditary disease*	7 (12.7%)		
AKI	6 (10.9%)		
Tubulointerstitial disease	4 (7.3%)		
Glomerular disease	3 (5.5%)		

^*^Including 4 ciliopathies, 1 Dent disease, 1 hyperuricemic nephropathy

Mann–Whitney test

Basic biochemical testing results are presented in Table 2. In the control group, kidney function was normal, with a median eGFR of 123.0 (112.2–148.4) ml/min/1.73 m^2^ and urea within the normal limit. Patients with CKD had significantly lower hemoglobin levels, with no other differences between the groups in full blood count parameters, nor the derived subclinical inflammation markers (NLR and PLR). Regarding the standard CKD-MBD markers, total calcium and parathyroid hormone were higher in the CKD group, but we found no differences in phosphate or alkaline phosphatase (ALP) levels. Within the lipid profile, patients with CKD presented higher total cholesterol and triglycerides. The acid–base parameters were comparable, and so were serum sodium and IGF-1. Serum potassium was higher in children with CKD. Of note, even though hemoglobin and serum potassium levels differed significantly between children with CKD and healthy controls, their median and IQR values remained within the normal limits in both groups.

**Table 2:** Laboratory test results in the examined groups

Parameter	Entire group	CKD	Control group	*p* value
NLR	1.2 (0.9–1.7)	1.4 (0.8–1.7)	1.1 (0.9–1.5)	0.608
PLR	109.2 (80.6–153.1)	103.9 (78.3–153.1)	112.6 (86.2–154.3)	0.580
White blood cells (10^3^/µl)	6.7 (5.5–8.2)	6.8 (5.4–8.2)	6.6 (5.6–9.1)	0.702
Lymphocyte count (10^3^/µl)	2.5 (1.9–3.1)	2.5 (1.8–2.9)	2.7 (2.2–3.3)	0.182
Neutrophil count (10^3^/µl)	2.9 (2.3–4.2)	2.9 (2.4–4.1)	2.9 (2.3–4.3)	0.964
Platelet count (10^3^/µl)	281.5 (223.0–336.0)	264.0 (206.0–324.0)	309.0 (251.0–356.0)	0.068
Hemoglobin [g/dl]	12.3 (11.4–12.9)	12.1 (11.3–12.9)	12.5 (11.9–13.4)	0.042
eGFR [ml/min/1.73 m^2^]	45.3 (20.4–107.3)	31.6 (18.3–49.2)	123.0 (112.2–148.4)	< 0.001
Urea [mg/dl]	65.5 (31.0–109.1)	75.8 (55.5–113.5)	26.5 (21.3–31.9)	< 0.001
Total calcium [mg/dl]	10.1 (9.9–10.4)	10.3 (9.9–10.5)	10.0 (9.8–10.1)	0.004
Phosphate [mg/dl]	4.9 (4.5–5.2)	5.0 (4.3–5.2)	4.9 (4.7–5.1)	0.724
ALP [IU/l]	257.0 (207.0–319.0)	257.5 (185.0–306.0)	246.0 (218.0–329.0)	0.482
PTH [pg/ml]	47.3 (28.3–133.0)	79.1 (45.2–194.0)	28.3 (14.0–36.9)	< 0.001
Total protein [g/dl]	7.1 (6.8–7.6)	7.2 (6.8–7.6)	7.0 (6.9–7.4)	0.378
Albumin [g/dl]	4.7 (4.5–4.8)	4.7 (4.5–4.8)	4.7 (4.6–4.9)	0.534
Total cholesterol [mg/dl]	174.6 (159.0–201.0)	185.5 (170.0–213.0)	160.0 (149.0–174.0)	< 0.001
Triglycerides [mg/dl]	91.0 (65.0–137.0)	114.0 (77.8–170.0)	67.0 (52.0–86.0)	< 0.001
Glucose [mg/dl]	86.0 (81.0–93.0)	87.0 (82.7–94.0)	83.0 (81.0–87.0)	0.061
Potassium [mmol/l]	4.5 (4.2–4.7)	4.5 (4.3–4.9)	4.3 (4.2–4.5)	0.032
Sodium [mmol/l]	138.0 (137.0–140.0)	138.0 (137.0–140.0)	138.0 (137.0–139.0)	0.489
Bicarbonate [mmol/l]	23.6 (22.2–25.5)	23.5 (21.8–25.5)	24.3 (23.0–25.7)	0.425
Base excess [mmol/l]	− 0.9 (− 2.5–1.1)	− 1.0 (–2.9–1.6)	− 0.7 (− 1.6–1.0)	0.593
IGF-1 [ng/ml]	126.0 (89.7–190.0)	126.0 (83.8–192.5)	133.0 (104.0–170.0)	0.602

*NLR* neutrophil-to-lymphocyte ratio, *PLR* platelet-to-lymphocyte ratio, *eGFR* estimated glomerular filtration rate, *ALP* alkaline phosphatase, *PTH* parathyroid hormone, *IGF-1* insulin-like growth factor 1

Mann–Whitney test

### Suppressor of cytokine signaling 2 (SOCS2)

**Fig. 1:**
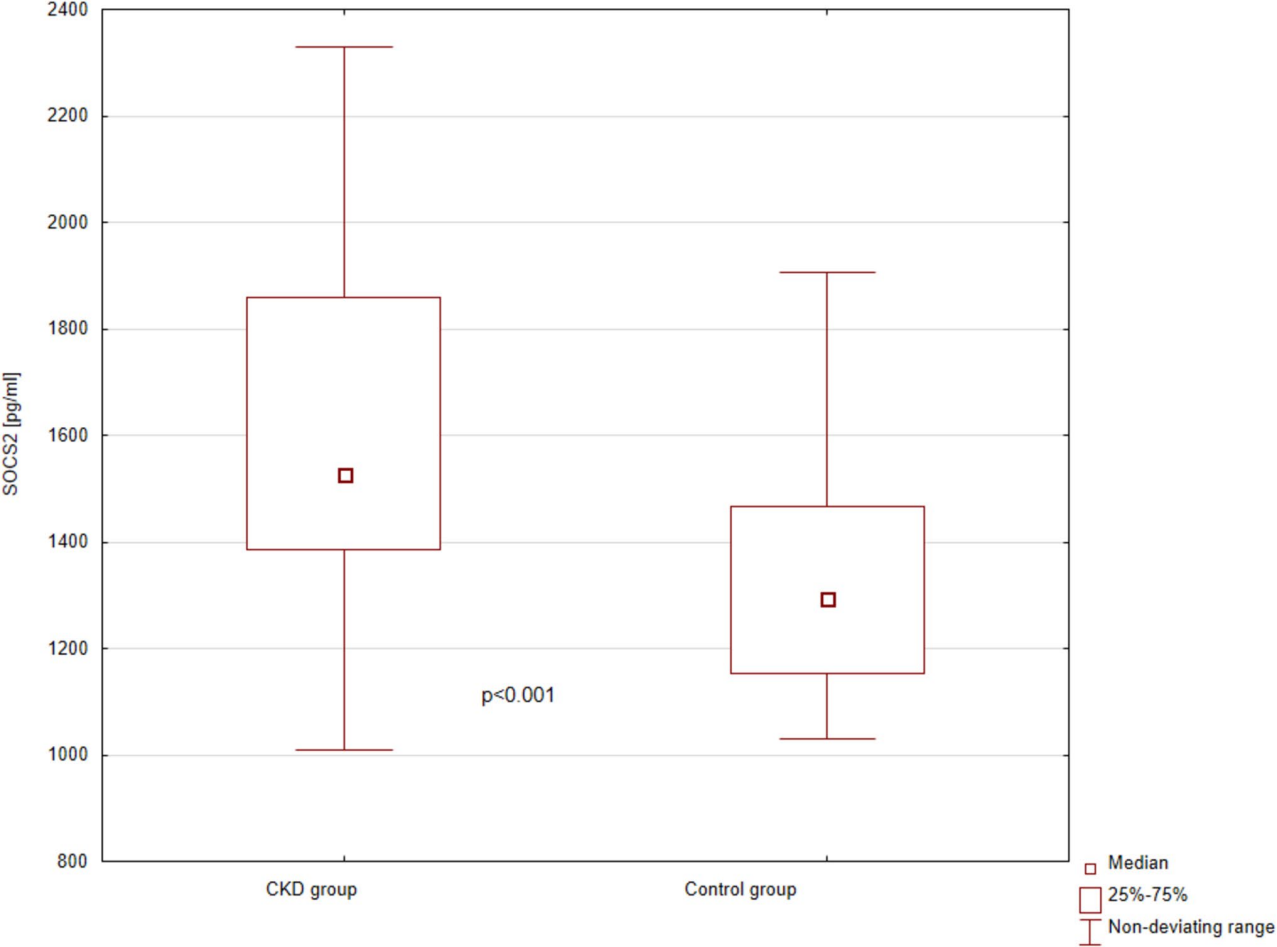
Serum suppressor of cytokine signaling 2 concentrations in patients with CKD and in healthy controls. Mann–Whitney test

SOCS2 was detectable in serum in all examined children, with a median concentration of 1458.5 (1284.7–1761.2) pg/ml. Its levels were significantly higher in patients with CKD compared to the control group (1526.5, IQR 1385.0–1860.0 pg/ml vs. 1294.6, IQR 1154.0–1468.0 pg/ml, respectively, *p* < 0.001) (Fig. 1). There were no differences in SOCS2 concentrations between girls and boys, in either the entire studied population (1449.8, IQR 1260.3–1837.4 pg/ml vs. 1462.7, IQR 1293.8–1719.2 pg/ml, *p* = 0.799), or in the CKD group (1626.5, IQR 1385.0–1860.0 pg/ml vs. 1493.4, IQR 1393.2–1855.6 pg/ml, respectively, *p* = 0.573). SOCS2 levels did not differ significantly between different primary kidney diseases (CAKUT 1465.4, IQR 1329.6–1740.2 pg/ml; cystic kidney diseases 1552.6, IQR 1400.7–1772.9 pg/ml; hereditary disease 2002.2, IQR 1580.3–2095.6 pg/ml; tubulopathies 1855.1, IQR 1626.6–2076.3 pg/ml; AKI 1639.2, IQR 1359.6–1867.6 pg/ml; glomerular diseases 1472.4, IQR 1299.5–1586.2 pg/ml; *p* = 0.127). We found no statistically significant correlations of SOCS2 concentrations with the patients’ age in either the entire population (*r* = 0.09, *p* = 0.447), or in the CKD group (*r* = 0.11, *p* = 0.417).

In the CKD group, we found a statistically significant negative correlation between SOCS2 levels and HtSDS (*r* =  − 0.30, *p* = 0.029), and positive correlations with neutrophil count (*r* = 0.30, *p* = 0.029) and neutrophil-to-lymphocyte ratio (*r* = 0.34, *p* = 0.013). There was no significant correlation between SOCS2 concentration and eGFR (*r* = 0.00, *p* = 0.987). In multivariate analysis, only HtSDS (*β* =  − 0.33, 95% CI − 0.59 to  − 0.06) correlated with SOCS2 levels.

### Growth impairment

In the examined population, median HtSDS was significantly lower in the CKD group (Table 1). HtSDS did not differ regarding primary kidney disease (*p* = 0.906). Among patients with CKD, 39 (70.9%) were short-statured. Importantly, this high ratio is related to the profile of our center (admitting CKD patients from the entire country for GH treatment qualification), and does not reflect the overall incidence of growth impairment in Polish CKD children. None of the participants were receiving GH treatment at the time of the study. None of the controls presented with growth impairment. The relatively high median HtSDS in the control group most probably results from the fact that Polish children (according to the most recent OLA-OLAF growth charts) are taller than the WHO population [15]. In this study, we use WHO-driven HtSDS for consistency.

**Fig. 2:**
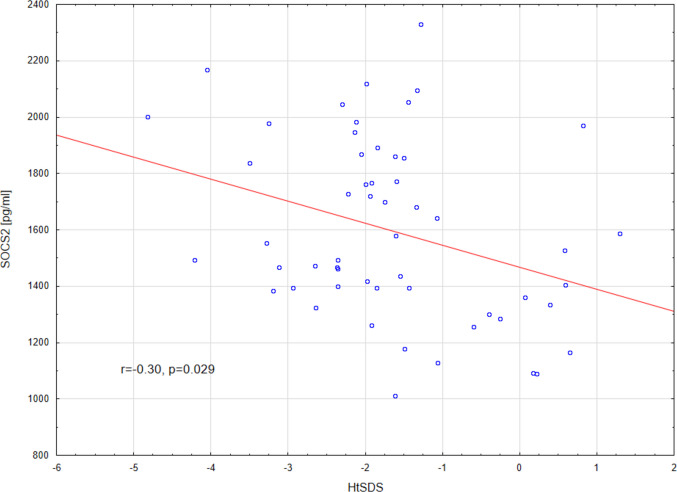
Correlation of serum SOCS2 concentrations with height SDS in the CKD group

In the CKD group, HtSDS correlated negatively with SOCS2 (*r* =  − 0.30, *p* = 0.029) and prematurity (r =  − 0.38, *p* = 0.004). We found positive correlations of HtSDS with age (*r* = 0.43, *p* = 0.001) and eGFR (*r* = 0.38, *p* = 0.007). The correlation of SOCS2 concentrations and HtSDS in the CKD group is displayed in Fig. 2. The multivariate analysis (general stepwise regression model) was adjusted for age, prematurity and past high-dose glucocorticoid use. In this model, eGFR (*β* = 0.43, 95% CI 0.21–0.65) and SOCS2 (*β* =  − 0.29, 95% CI − 0.51 to − 0.07) were the predictors of HtSDS. Adjusted *R*^2^ of the model was 0.430. Interestingly, the general regression model built that included the same parameters but without SOCS2 also identified eGFR as the predictor but had lower adjusted *R*^2^ (0.377).

**Fig. 3:**
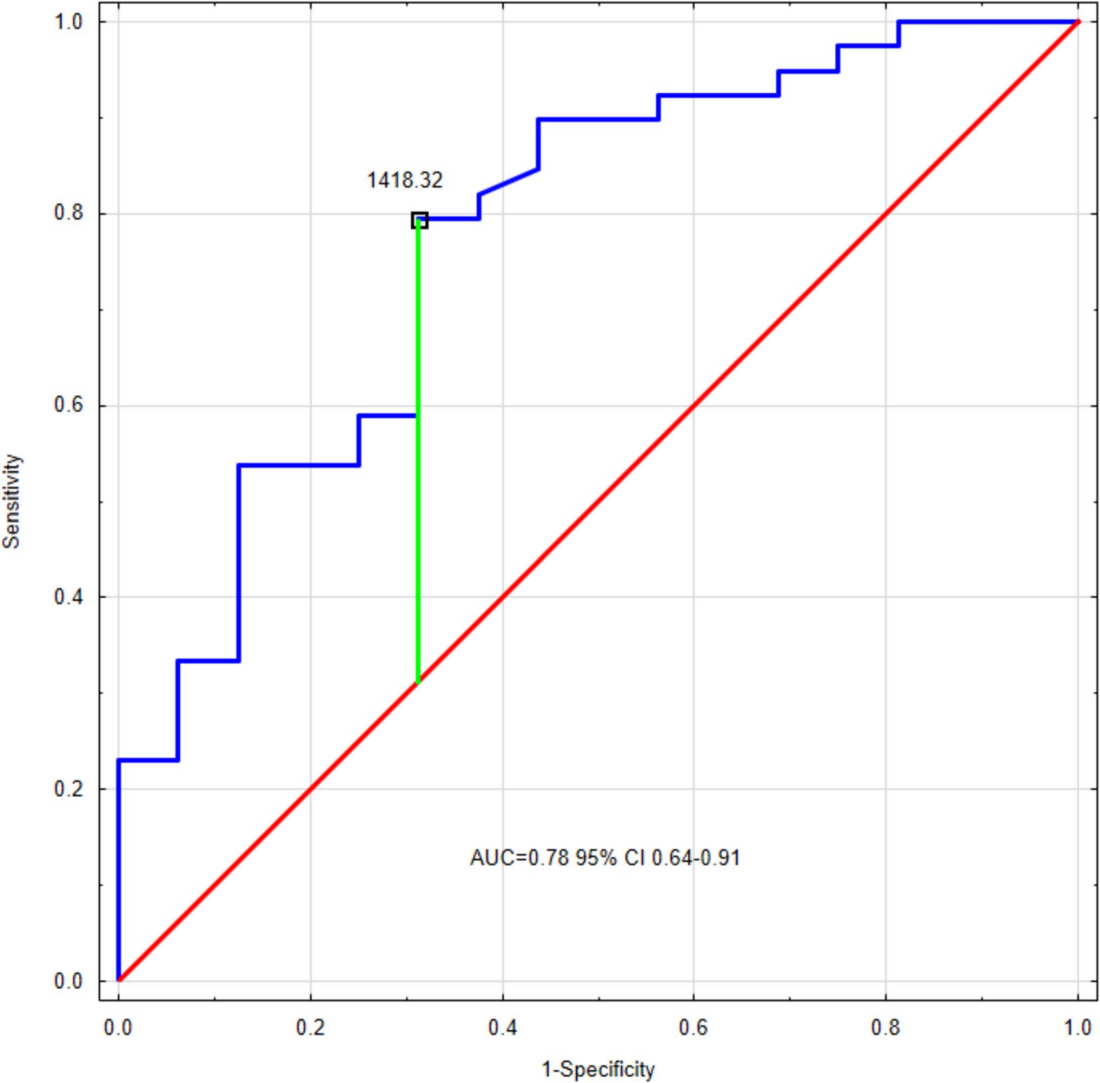
ROC curve for SOCS2 predicting the occurrence of short stature within the CKD group

Within the CKD group, in short-statured children, SOCS2 levels were higher than in patients without height deficit (1698.2, IQR 1435.1–1946.6 pg/ml vs. 1347.0, IQR 1210.0–1556.4 pg/ml, respectively, *p* < 0.001). ROC analysis demonstrated a moderate-to-good diagnostic profile for predicting short stature (AUC 0.78, 95% CI 0.64–0.91, *p* < 0.001). The SOCS2 cut-off value of 1418.32 pg/ml predicted growth deficit with 79.5% sensitivity and 68.8% specificity (Fig. 3).

## Discussion

To the best of our knowledge, the present study is the first one to demonstrate that serum SOCS2 levels are significantly elevated in children with CKD compared to healthy controls. Furthermore, we identified a negative correlation between SOCS2 concentration and HtSDS, suggesting that SOCS2 may be associated with growth impairment in pediatric CKD.

SOCS2 functions as a negative regulator of the JAK-STAT5b signaling pathway, which plays a vital role in supporting normal growth and development [18]. In children with CKD, suppression of the JAK/STAT5b pathway has been shown to contribute to impaired GH signaling [19]. In murine studies, deletion of the *Socs2* gene led to excessive growth and heightened sensitivity to growth hormone (GH) [6, 8]. In contrast, homozygous mutations in *STAT5b* have been linked to growth failure and GH insensitivity in humans [20–23]. Moreover, children with idiopathic short stature (ISS) showed elevated SOCS2/3 expression, which increased further with GH stimulation, suggesting a potential role of SOCS overexpression in their growth impairment [24]. Our observation of elevated SOCS2 expression in pediatric CKD aligns with prior findings from animal models. For instance, increased *Socs2* expression has been reported in immature rats with CKD [25]. More recently, it was shown that *Socs2*-deficient mice with CKD are able to phosphorylate STAT5 and maintain normal growth, whereas *Socs2*-positive mice exhibit growth disturbances [26]. These animal studies are particularly relevant to pediatric CKD, where peripheral GH resistance is a well-established contributor to growth retardation [27].

The elevated levels of SOCS2 in children with CKD compared to healthy controls may be associated with inflammatory processes. Subclinical chronic inflammation is a common consequence of CKD [28]. Previous studies have shown that SOCS2 is produced in response to GH stimulation, but also to increasing levels of proinflammatory cytokines (i.e., tumor necrosis factor-α—TNF-α and interleukin-6—IL-6) [29], which are elevated in CKD [30, 31]. Although classic markers of inflammation such as NLR and PLR were not elevated in our cohort, we observed positive correlations between SOCS2 and NLR, and neutrophil count, which might suggest a potential influence of subclinical inflammation.

The observed inverse correlation between SOCS2 and HtSDS underlines the potential role of this protein in growth impairment. Importantly, SOCS2 remained independently associated with HtSDS in multivariate analysis alongside a known predictor—eGFR [1]—improving the *R*^2^ of the model. This finding suggests that SOCS2 may provide additional explanatory power for models predicting growth, beyond traditional clinical markers.

Our ROC analysis demonstrated acceptable-to-good diagnostic performance. Sensitivity was moderate-to-good, and specificity was moderate. This suggests that circulating SOCS2 might complement the traditional markers in identifying children at greatest risk for growth impairment. However, longitudinal studies on larger populations are required to confirm this. Of note, ROC diagnostic performance may have been negatively influenced by a relatively small number of short-statured children.

Notably, the majority of biochemical parameters in the CKD cohort were comparable to those of healthy controls, suggesting a relatively stable metabolic state among patients in this cohort. Even though PTH and lipid profile parameters were higher in patients with CKD, none of these parameters emerged as a HtSDS predictor in multivariate models. These observations reduce the possibility that observed differences in SOCS2 levels were driven by metabolic imbalance resulting from CKD. Elevated SOCS2 levels observed in this study may point to a more direct role in growth suppression, potentially mediated through low-grade inflammation. This supports the possibility of considering SOCS2 as an independent marker, or a contributor, in CKD-related growth failure, even in clinically stable patients.

This study presents a novel approach in evaluating circulating SOCS2 levels in children, and is the first one to show that SOCS2 levels are higher in children with CKD than in their healthy peers. However, it has several limitations. The cross-sectional design does not enable us to conclude causal connections between the variables. Longitudinal studies are needed to draw conclusions on the potential cause-and-effect relationship between SOCS2 and growth retardation. The high prevalence of short stature resulted from the center profile and does not reflect the overall epidemiology in Polish CKD patients. The SOCS2 ELISA kit used is relatively new, and although validated by the manufacturer (Assay Genie), requires further standardization across laboratories. We excluded patients with growth retardation of a different origin than CKD, but factors such as socioeconomic status or unmeasured endocrine parameters might have influenced results. Finally, the relatively small sample size limits subgroup analysis, especially between different CKD stages and etiologies.

Although our findings are not immediately translatable into clinical decision-making, they might carry potential implications for practice in the long term. Elevated SOCS2 levels, if confirmed in larger and longitudinal studies, could complement established clinical parameters in identifying children with CKD who are at higher risk of growth impairment. Furthermore, future research might clarify whether SOCS2 assessment could contribute to tailoring growth hormone treatment strategies.

## Conclusion

Our findings suggest that circulating SOCS2 is elevated in children with CKD and is independently associated with growth impairment. These results provide new insight into the mechanisms underlying GH resistance in pediatric CKD and suggest that SOCS2 may serve as a useful biomarker of growth impairment. Our future efforts will focus on longitudinal studies to determine if SOCS2 levels change in response to GH treatment and whether they influence recombinant GH treatment response.

## Supplementary Information

Below is the link to the electronic supplementary material.

ESM 1 Graphical abstract  (93.2 KB)

ESM 2 (DOCX 102 KB)

## Data Availability

The datasets generated and analyzed during the current study are available from the corresponding author on reasonable request.
