# Supplementary material for: Suppressor of cytokine signaling 2 is associated with growth impairment in pediatric chronic kidney disease
Source: Pediatr Nephrol. 2025 Nov 28;41(4):1151–9. doi: 10.1007/s00467-025-07029-0 (PMC12953468; doi:10.1007/s00467-025-07029-0)
Supplement: Supplementary file 1 — Graphical abstract (93.2 KB) [file 467_2025_7029_MOESM1_ESM.pptx]

## Slide 1
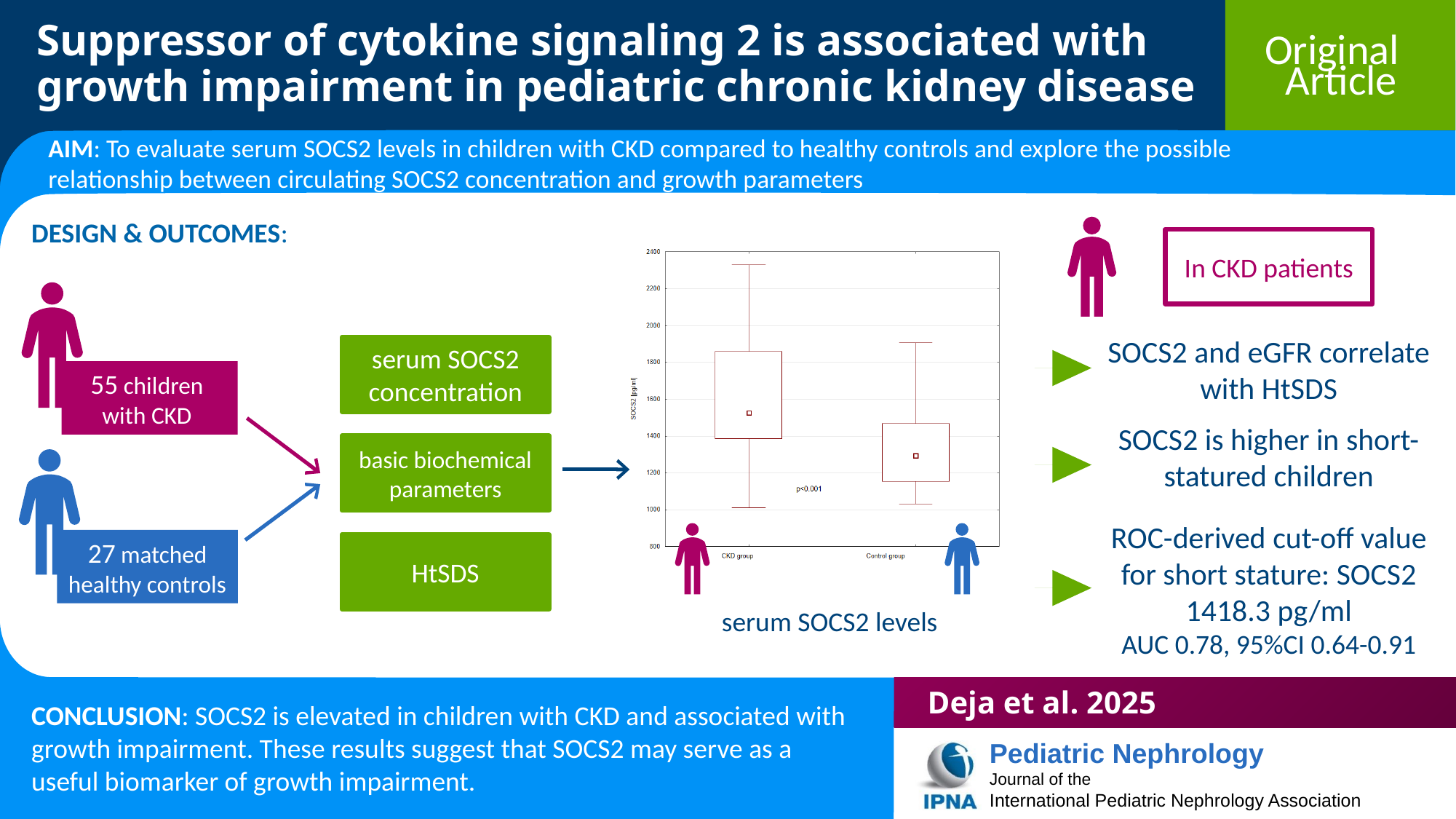

Suppressor of cytokine signaling 2 is associated with growth impairment in pediatric chronic kidney disease
AIM: To evaluate serum SOCS2 levels in children with CKD compared to healthy controls and explore the possible relationship between circulating SOCS2 concentration and growth parameters
DESIGN & OUTCOMES:
In CKD patients
55 children
with CKD
SOCS2 and eGFR correlate with HtSDS
serum SOCS2 concentration
basic biochemical parameters
HtSDS
SOCS2 is higher in short-statured children
27 matched healthy controls
ROC-derived cut-off value for short stature: SOCS2 1418.3 pg/ml
AUC 0.78, 95%CI 0.64-0.91
serum SOCS2 levels
Deja et al. 2025
CONCLUSION: SOCS2 is elevated in children with CKD and associated with growth impairment. These results suggest that SOCS2 may serve as a useful biomarker of growth impairment.
