# Supplementary material for: Suppressor of cytokine signaling 2 is associated with growth impairment in pediatric chronic kidney disease
Source: Pediatr Nephrol. 2025 Nov 28;41(4):1151–9. doi: 10.1007/s00467-025-07029-0 (PMC12953468; doi:10.1007/s00467-025-07029-0)
Supplement: Supplementary file 2 — (DOCX 102 KB) [file 467_2025_7029_MOESM2_ESM.docx]

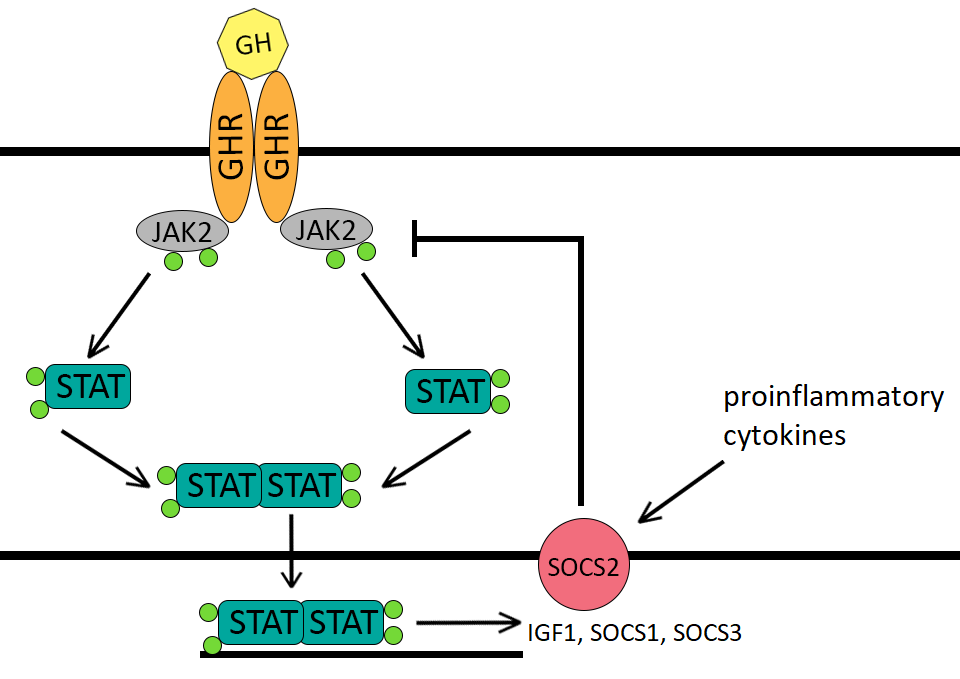


**Supplementary figure 1** Activation of the JAK-STAT pathway.


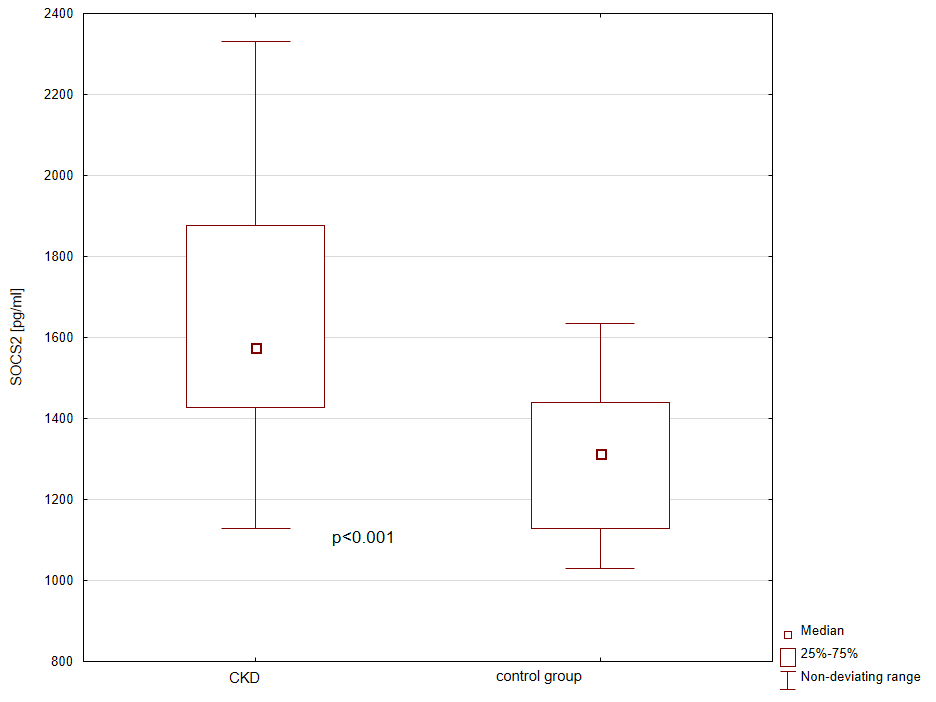


**Supplementary figure 2** Results of the preliminary study, conducted to assess SOCS2 serum detectability and potential differences between children with CKD and healthy age- and sex-matched controls. Comparison of serum SOCS2 concentrations in CKD (1576.1, IQR 1427.2-1876.4 pg/ml, n=20) and control groups (1314.1, IQR 1129.5-1438.8 pg/ml, n=20).
